# Chinese Medicines as an Adjuvant Therapy for Unresectable Hepatocellular Carcinoma during Transarterial Chemoembolization: A Meta-Analysis of Randomized Controlled Trials

**DOI:** 10.1155/2013/487919

**Published:** 2013-07-17

**Authors:** Fan Cheung, Xuanbin Wang, Ning Wang, Man-Fung Yuen, Tat-chi Ziea, Yao Tong, Vivian Taam Wong, Yibin Feng

**Affiliations:** ^1^School of Chinese Medicine, LKS Faculty of Medicine, The University of Hong Kong, Hong Kong; ^2^Laboratory of Chinese Herbal Pharmacology, Renmin Hospital and School of Pharmacy, Hubei University of Medicine, Hubei 442000, China; ^3^Department of Medicine, LKS Faculty of Medicine, The University of Hong Kong, Queen Mary Hospital, Pokfulam Road, Hong Kong; ^4^Chinese Medicine Department, Hospital Authority, Hong Kong

## Abstract

*Objective*. To conduct a comprehensive PRISMA-compliant systematic review and meta-analysis to evaluate the efficacy and safety of Chinese medicines (CMs) as an adjuvant therapy for unresectable HCC during transarterial chemoembolization (TACE). *Methods*. Main databases were searched up to October 2012 for randomized controlled trials (RCTs) evaluating the effects of CMs plus TACE on unresectable HCC compared with TACE alone. References of relevant reviews and eligible studies were also assessed. Risk ratios with 95% confidence intervals and mean difference were calculated. Heterogeneity and publication bias were examined. *Results*. Sixty-seven trials (*N* = 5,211) were included in the meta-analysis. Sensitivity analysis and random-effects model were performed for assessing significant heterogeneity. CMs plus TACE showed beneficial effects on tumor response, survival at 6, 12, 18, 24, and 36 months, quality of life, and TACE toxicity reduction compared with TACE alone. *Conclusion*. The results show that the use of CMs may increase the efficacy and reduce the toxicity of TACE in treating patients with unresectable HCC. These findings suggest that CMs could be considered as an adjuvant therapy for unresectable HCC patients during TACE. Larger-scale RCTs using standard methods and long-term follow-up are warranted to confirm these findings.

## 1. Introduction

Liver cancer, mainly hepatocellular carcinoma (HCC), ranks the sixth most common cancer and the third leading cause of cancer-related death worldwide [1, 2]. Annually, more than 748,000 new cases are diagnosed and 695,000 died with liver cancer. HCC is mostly unresectable as many were detected at advanced stage with poor liver function, high tumor recurrence rate, and metastasis [3]. As most HCC patients are not suitable candidates for curative resection, transarterial chemoembolization (TACE) is the most commonly used for unresectable HCC patients as a primary and palliative therapy because of improvement in survival [4–6]. However, severe side effects including liver and renal failure, bone marrow depression, postembolization syndrome, and liver abscess were observed with the use of TACE [4, 7].

Chinese medicines (CMs) were commonly used in treating HCC with side effects seldom reported. Increasing number of studies was conducted in assessing the effects of CMs on HCC. Experimental studies found the chemopreventive effects and anti-HCC properties of CMs mainly through the induction of apoptosis and autophagy and cytotoxicity on cancer cells [75–78]. Although three systematic reviews evaluating the efficacy of CMs on HCC had been published [79–81], the effect of CMs combined with TACE in treating HCC remains uncertain. No systematic review was conducted according to the preferred reporting items for systematic reviews and meta-analyses (PRISMA) [82]. Moreover, two of these reviews included nonrandomized controlled trials which probably overestimated the beneficial effects of CMs [79, 81]. Another review had not focused on specific stage of HCC [80]. In addition, a significant proportion of related randomized controlled trials (RCTs), especially those published recently (2007 afterward, 27 studies), were not included in these reviews. Therefore, we conducted a comprehensive and PRISMA-compliant systematic review and meta-analysis to investigate the efficacy of CMs on unresectable HCC including updated trials published after 2007. Specifically, we aim to critically appraise the efficacy and safety of CMs as an adjuvant therapy for unresectable HCC patients during TACE treatment focusing on outcomes of survival, tumor response, quality of life (QoL), and TACE toxicity. 

## 2. Methods

This systematic review was conducted according to the PRISMA statement [82].

### 2.1. Search Strategies

Main electronic databases including MEDLINE (1946–2012), EMBASE (1947–2012), AMED (1985–2012), CINAHL Plus (1937–2012), PubMed (January 1966–2012), the Cochrane Library (1996–2012), Chinese Biomedical CD Database (CBM, January 1980–2012), China Network Knowledge Infrastructure (CNKI, 1911–2012), TCMOnline (1949–2012), Chinese Medical Current Contents (CMCC, 1994–2012), and WanFang Data (1989–2012) were searched for eligible studies. The latest search was performed on October 2012. References of relevant reviews and eligible studies were also checked. 

The search terms used were “liver cancer,” “hepatocellular carcinoma,” “primary liver carcinoma,” “Chinese medicine,” “herbal medicine,” “traditional medicine,” and “complementary medicine” without restriction on publication language and publication type. Free-text and MeSH terms were used when allowed. The search strategies in Chinese and English were slightly adjusted to suit the instructions of different databases.

### 2.2. Study Selection Criteria

Eligible RCTs examining the efficacy of CMs plus TACE in treating unresectable HCC were assessed. Inclusion criteria were as follows: (a) RCTs; (b) participants in treatment group received combination therapy consisting of CMs and TACE and TACE alone in control group; (c) participants had unresectable or stage II or above primary HCC which were confirmed by cytological or pathological results, or met the criteria of the European association for the study of the liver guideline; (d) reported data on at least one of the outcomes including survival, tumor response, QoL using the Karnofsky performance scale (KPS), or TACE-related toxicity.

Primary outcomes were 6-month, 12-month, 18-month, 24-month, and 36-month survival and tumor response. Secondary outcomes included KPS (QoL) and TACE toxicity. Survival was defined as the number of patients in each intervention group who were alive at 6, 12, 18, 24, or 36 months. Tumor response has to be assessed using the World Health Organization (WHO) criteria, which were commonly used to evaluate therapeutic efficacy on solid tumors [83, 84]. According to the results of CT and/or MRI, the efficacy of anticancer agents was classified as follows: complete response (CR) refers to the disappearance of all visible tumor lesions; partial response (PR) refers to 50% or more decrease in the lesions; no change (NC) refers to either less than 50% decrease in total tumor size or at least 25% increase in the lesions; and progressive disease (PD) refers to at least 25% increase in the size of the lesions. Tumor response was defined as CR plus PR and compared before and after treatment. TACE-related toxicity including gastrointestinal and bone marrow toxicities was evaluated using the 5-point WHO scale (grade 0–4) on reporting acute and subacute toxic effects [85]. 

Exclusion criteria included the following: (a) using other complementary medicines in treatment or control group; (b) metastatic HCC; (c) inconsistency of reporting on methods, results, or both; and (d) duplicated or redundant publications. 

### 2.3. Study Selection

All searched titles and abstracts were screened independently by two authors (Fan Cheung and XuanbinWang) according to the predefined eligibility criteria. Disagreements were resolved by consensus or consulting a third author (Yibin Feng). Full texts of the potentially eligible studies were retrieved and further assessed by these two authors (Fan Cheung and XuanbinWang) using the same method.

### 2.4. Data Extraction

Data of the included studies were extracted independently and cross-checked by two authors (FC and XBW) using a standardized extraction form which was generated at the protocol stage. The extracted items comprised (1) authors and year of publication; (2) study design; (3) participant characteristics; (4) intervention details, and (5) outcome measures. 

### 2.5. Study Quality Assessment

Study quality was independently evaluated by two authors (Fan Cheung and XuanbinWang) using the six dimensions of Cochrane “risk of bias” assessment [114]. The assessment criteria included sequence generation, allocation concealment, blinding, incomplete outcome data, selective outcome data, and other bias. Each dimension was rated as “yes” (low risk of bias), “unclear” (unclear risk of bias), or “no” (high risk of bias). Studies with 3 or more “yes” were classified as high quality with low risk of bias and 0–2 poor quality with high risk of bias. As bias of blinding may be more severe for subjective outcomes (e.g., QoL) than for objective outcomes (e.g., survival and tumour response), separate analyses for different outcomes were conducted as recommended by Cochrane collaboration [114]. 

### 2.6. Statistical Analysis

Review Manager 5.1 (The Nordic Cochrane Centre, Copenhagen, Denmark) was used for data analysis. Risk ratios (RRs) with 95% confidence intervals (CIs) and mean difference (MD) were calculated for dichotomous and continuous data, respectively. Heterogeneity was assessed using *X* ^2^ test and *I* ^2^ statistic with *P* < 0.1 or *I* ^2^ > 50% was treated as substantial heterogeneity [114]. Significant statistical heterogeneity was further assessed using sensitivity analyses and results were estimated using random-effects model. In contrast, a fixed-effect model was used for homogeneous studies. Publication bias was examined using funnel plots [115] and Egger's test [116] (STATA 10.0, StataCorp LP, College Station, TX, USA). *P* values lower than 0.05 were considered statistically significant. 

## 3. Results

**Figure 1:**
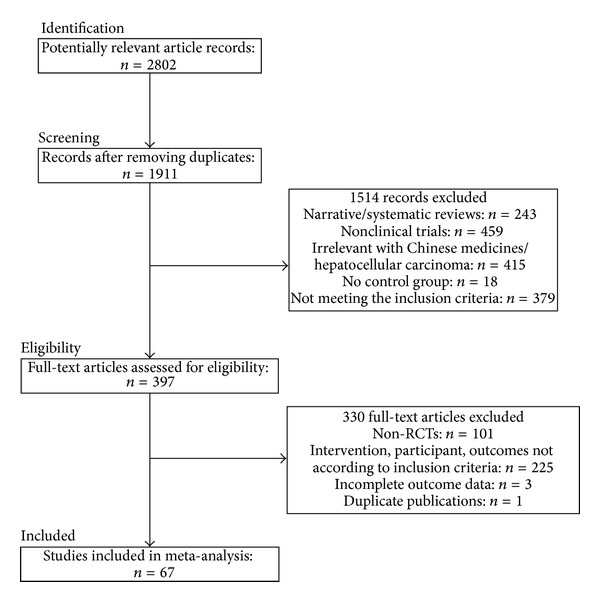
Flow diagram of the study selection for this systematic review.

A total of 2802 potential trials were identified for this review, of which 891 were duplicate records and 1514 were excluded because of narrative/systematic review, nonclinical trials, irrelevance, no comparison group, or not meeting the inclusion criteria of this study (Figure 1). The full text of 397 articles was retrieved for further evaluation, of which 330 were excluded for the reasons of not RCTs (*n* = 101), not according to the inclusion criteria (*n* = 255), incomplete outcome data (*n* = 3), or duplicate publication (*n* = 1). Finally, 67 RCTs with a total of 5211 patients (study sample size ranged from 25 to 236) [8–74] were included in this study. Two of the included studies were retrieved from the relevant reviews and studies [20, 21]. 

### 3.1. Study Descriptions

All studies were conducted in hospital settings in China, of which 6 were multicentre studies [18, 36, 39, 57, 58, 67] and the remaining were single-centre studies (Table 1). All studies adapted parallel-arm group design. Nearly, all studies, except one [18], were published in Chinese from 1999 to 2011. Participants aged from 18 to 78 years old. Near half (*n* = 32) described the enrollment criteria (diagnosis, inclusion and exclusion criteria). 

Three studies used individualized prescriptions according to traditional CM syndrome patterns [17, 63, 73], while 46 standardized CM formulae including 4 single herbs and 42 composite formulae were tested in the remaining 64 studies. Ai Di injection (*n* = 8) was the most popularly used standardized CM formula. The duration of CMs treatment ranged from 14 days to 3 years.

### 3.2. Methodological Quality

Of the 67 included studies, only 15 studies reported the methods of allocation sequence generation, which included using a random number table [11, 26, 43, 60, 65], drawing of lots [17], shuffling envelops [28, 56], stratified randomization [30, 63], and referring to the sequence of admission [12, 49, 64, 69, 70]. The remaining 52 studies described that the participants were “randomly allocated,” but the allocation procedures were not reported. None of the studies mentioned the method of allocation concealment. Twenty studies reporting objective outcomes were rated as at low risk of blinding bias. Most studies (82%) reported no significant difference of baseline characteristics. No study described intention-to-treat analysis. Only 6 studies [21, 38, 47, 59, 60, 65] reported the information of dropouts, in which 3 studies [21, 38, 59] provided reasons of withdrawal. Forty studies were rated as at low risk of bias for incomplete outcome reporting, 5 (7%) at high risk, and 22 could not be rated due to insufficient information. Thirty-nine studies (58%) were rated as at low risk of bias for selective outcome data, 3 (4%) at high risk, and 25 (37%) did not provide sufficient information to permit judgment. Consequently, 29 studies were assigned as high quality with a low risk of bias (Table 2).

### 3.3. Meta-Analysis of Primary Outcomes

#### 3.3.1. Tumor Response (Short-Term Effectiveness)

**Figure 2:**
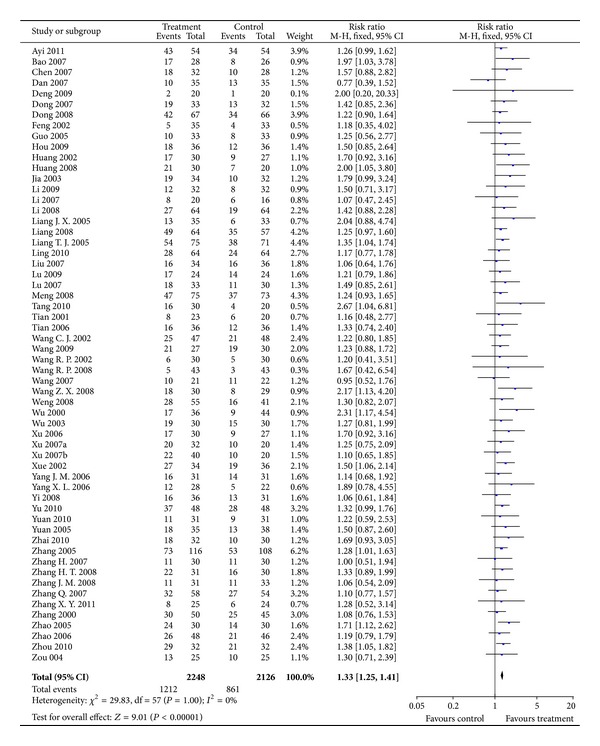
Results of Forest plots of comparison of CMs plus TACE versus TACE alone on tumor response (complete response + partial response) for HCC patients at middle and late stages. M-H: Mantel-Haenszel estimates; CI: confidence interval; CMs: Chinese medicines; TACE: transcatheter arterial chemoembolization.

Fifty-eight RCTs involving 4482 participants reported tumor response as an outcome for testing the short-term effect of CMs plus TACE (combination therapy). The combination therapy was found to be superior to TACE alone in increasing the short term effectiveness (RR = 1.33; 95% CI = 1.25 to 1.41; *P* < 0.00001) (Figure 2). The fixed-effect model was used to combine the data, whereas both *X* ^2^ and *I* ^2^ test suggested a low risk of heterogeneity (*P* = 1.00; *I* ^2^ = 0%). 

#### 3.3.2. Survival (Long-Term Effectiveness)

**Figure 3:**
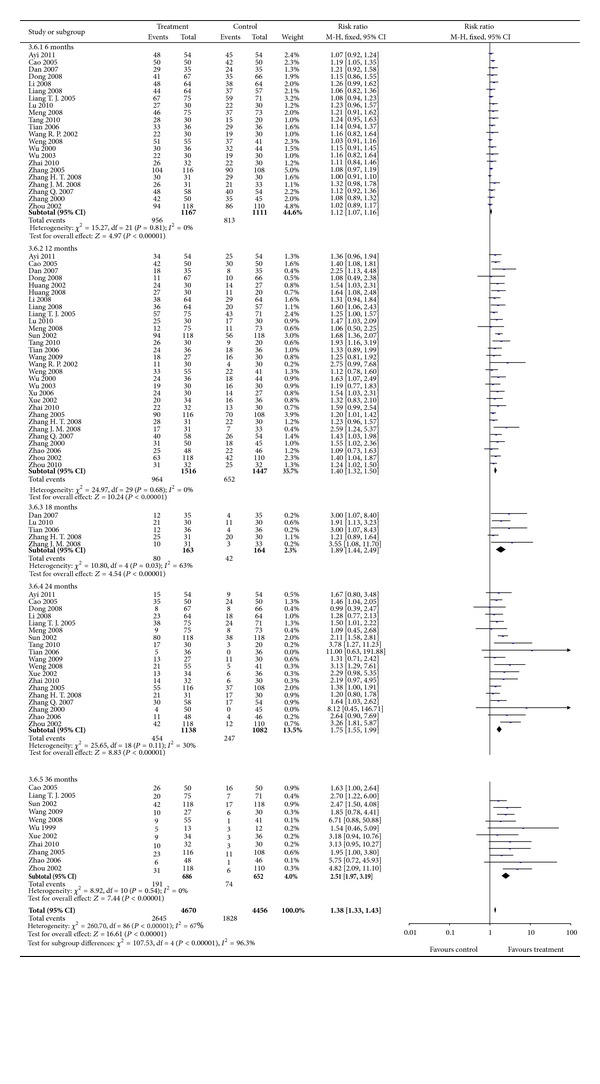
Results of Forest plots of comparison of CMs plus TACE versus TACE alone on 6-month, 18-month, 24-month, and 36-month survival for HCC patients at middle and late stages. M-H: Mantel-Haenszel estimates; CI: confidence interval; CMs: Chinese medicines; TACE: transcatheter arterial chemoembolization.

Thirty-two trials presenting 3038 participants reported the number of patients surviving for 6 to 60 months. Survival at 48 and 60 months were not evaluated as only 1 study [45] reported the results on this. Significant increases of survival at 6, 12, 18, 24, and 36 months for combination therapy were found with corresponding RRs (95% CI) of 1.12 (1.07 to 1.16), 1.39 (1.31 to 1.48), 1.89 (1.44 to 2.49), 1.75 (1.55 to 1.97), and 2.51 (1.97 to 3.19), all *P* < 0.00001 (Figure 3). The results were homogenous although significant heterogeneity was observed for survival at 18 months (*P* = 0.03; *I* ^2^ = 63%). However, similar estimates (RR = 2.52; 95% CI = 1.67 to 3.82; *P* < 0.0001) and homogeneity (*P* = 0.66; *I* ^2^ = 0%) were observed in sensitivity analysis by excluding a study with outlier. 

### 3.4. Meta-Analysis of Secondary Outcomes

#### 3.4.1. KPS

**Figure 4:**
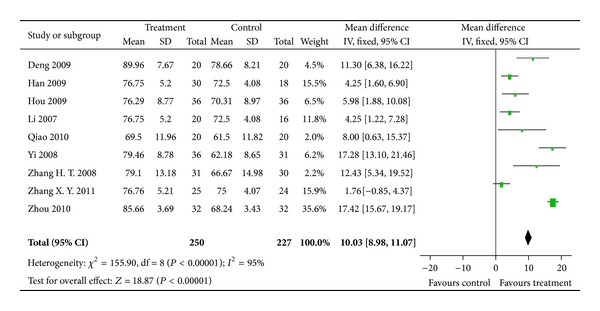
Results of Forest plots of comparison of CMs plus TACE versus TACE alone on Karnofsky score (continuous data) for HCC patients at middle and late stages. M-H: Mantel-Haenszel estimates; CI: confidence interval; CMs: Chinese medicines; TACE: transcatheter arterial chemoembolization.

KPS was measured in 36 studies for assessing the effect on QoL, in which continuous data was reported in 9 studies (*n* = 477 participants) and dichotomous data (KPS > 10) reported in 27 studies (*n* = 2041 participants). Significant differences in favor of combination therapy were found for continuous outcome of KPS (MD = 9.12; 95% CI = 4.17 to 14.07) (Figure 4). Random-effects model was used as heterogeneity was observed (*P* < 0.00001,   *I* ^2^ = 95%). Heterogeneity was reduced (*P* = 0.03, *I* ^2^ = 18%) after excluding studies with outliers and the significant difference between treatment and control groups was robust (RR = 3.94; 95% CI = 2.30 to 5.59; *P* < 0.00001). 

**Figure 5:**
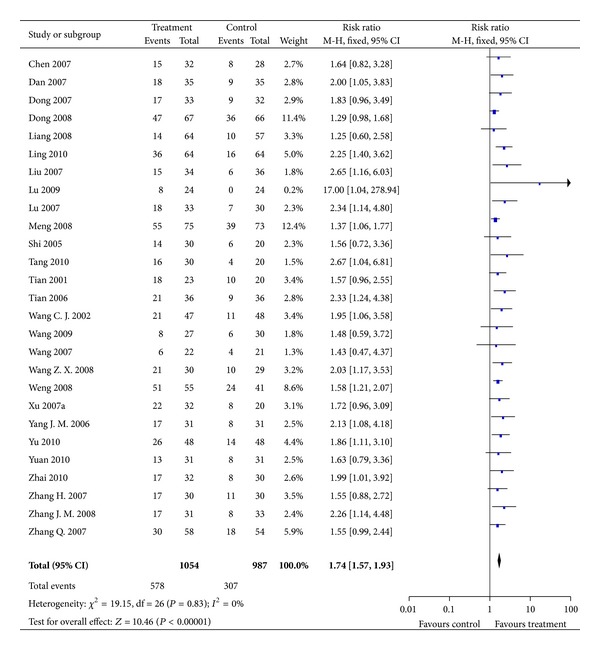
Results of Forest plots of comparison of CMs plus TACE alone versus TACE on KPS increased >10 points for HCC patients at middle and late stages. M-H: Mantel-Haenszel estimates; CI: confidence interval; CMs: Chinese medicines; TACE: transcatheter arterial chemoembolization; KPS: karnofsky performance score.

KPS >10 indicated that the results of KPS increased more than 10 points after treatment. A superior effect on the improvement of QoL in combination therapy compared with TACE alone was observed (RR = 1.74; 95% CI = 1.57 to 1.93; *P* < 0.00001) (Figure 5). As the result was homogenous (*P* = 0.83; *I* ^2^ = 0%), fixed-effect model was used.

#### 3.4.2. Reduction in TACE Toxicity (Short-Term Effectiveness)

**Figure 6:**
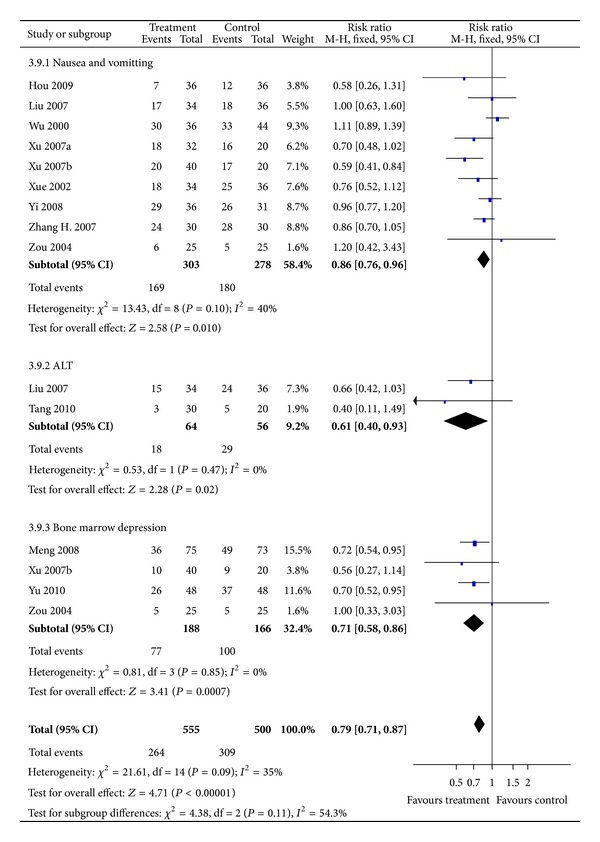
Results of Forest plots of comparison of CMs plus TACE versus TACE alone on TACE toxicity (grade 1–4) for HCC patients at middle and late stages. M-H: Mantel-Haenszel estimates; CI: confidence interval; CMs: Chinese medicines; TACE: transcatheter arterial chemoembolization.

Results of fixed-effect model in 12 studies showed that TACE toxicity including nausea and vomiting, alanine transaminase (ALT) elevation, and bone marrow depression were significantly reduced in treatment groups compared with TACE alone with corresponding RRs (95% CI) of 0.86 (0.76 to 0.96), 0.61 (0.04 to 0.93), and 0.71 (0.58 to 0.86) (Figure 6). Heterogeneity was not observed in the analysis (*P* = 0.1, 0.47, 0.85; *I* ^2^ = 40%, 0%, 0%; resp.). No chronic adverse reaction was reported in the studies.

#### 3.4.3. CMs-Related Side Effects

CMs-related side effects were rarely reported. Only 3 studies (4%) [20, 60, 74] reported low-grade fever (2 cases), dizziness (1 case), gastrointestinal discomfort (28 cases), and mild skin itch and rashes (3 cases). These symptoms were generally alleviated or recovered after symptomatic treatment. No severe side effects associated with CMs were reported in the included trials. The long-term side effects of the treatment were uncertain as only short-term effects were measured.

### 3.5. Risk of Bias across Studies

**Figure 7:**
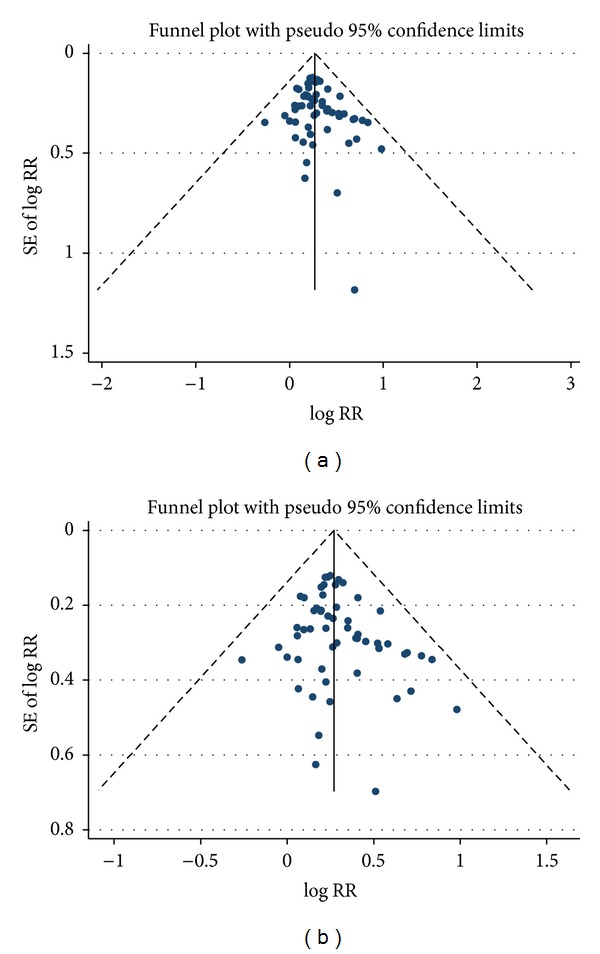
Funnel plots of (a) tumor response of the included studies and (b) tumor response of the included studies excluding a study with outlier results.

Risk of publication bias was assessed using funnel plot to compare symmetry for all studies except for one with outliers (Figure 7). Results of Egger's test suggested no significant publication bias of the included studies (*t* = 1.99,   *P* = 0.051). 

### 3.6. Common Herbs

The top 10 most frequently used herbs in the included trials were listed in Table 3 together with the potential pharmacological properties. Although the constituents of the formulae were varied across the trials, there was a general consensus in diagnosis based on unique Chinese medicine theory. Reinforcing healthy Qi and blood, clearing fire toxin, and resolving dampness were the most concerned therapeutic principles which were associated with improvement in short-term and long-term effectiveness. Radix Astragali (Huang Qi) (*n* = 35) was the most frequently used herb in the trials, followed by Poria Cocos, Rhizoma Atractylodis Macrocephalae, Radix Ginseng, Radix Bupleuri, Radix Codonopsis, Semen Coicis, Herba Oldenlandia Diffusa, Radix Paeoniae Alba, and Rhizoma Curcumae. These 10 herbs are worthy of additional investigation to examine the possible active components for the use in HCC treatment.

## 4. Discussion

TACE is one of the few therapeutic treatments for unresectable HCC patients. Although CMs are increasingly used to enhance the treatment effects of TACE and reduce the side effects, the effectiveness is uncertain as the updated evidence has not been systematically summarized.

This is the first PRISMA-compliant systematic review and meta-analysis for examining the efficacy of CMs plus TACE (combination therapy) in treating unresectable HCC patients. Sixty-seven studies involving 5,211 participants met the study selection criteria. The meta-analysis showed that the combination therapy was significantly better than TACE alone in increasing tumor response, prolonging survival, improving QoL, and reducing TACE toxicity. CM-related side effects were mild and rarely reported in the studies. The above findings suggested that CMs might play a potentially beneficial role in assisting TACE therapy to improve tumor response, survival, and QoL, as well as reduction in TACE toxicity. 

The results are robust as the analyses were based on 67 RCTs with a large sample size of more than 5000 subjects, and both short-term (tumor response, QoL, and TACE toxicity reduction) and long-term (survival) effectiveness were assessed. Although most of the included studies (*n* = 66) were published in Chinese, the trials in this review represent the best available evidence on the efficacy of CMs as an adjuvant therapy for unresectable HCC patients during TACE treatment. Moreover, this review was conducted using comprehensive, rigorous, and PRISMA-compliant methods. An extensive search was conducted for RCTs published before October 2012. Between-study heterogeneity was further assessed by sensitivity analysis and random-effects model. Publication bias was investigated by both visual funnel plots and Egger's test. 

Considerable variety in the ingredients of the CM formulae was found in this review which might be due to different TCM diagnosis and the CM practitioners' personal experience. However, a common consensus in TCM diagnosis and treatment principle was observed among the included trials. According to our review, CM herbs might enhance the tumor response by inhibiting tumor angiogenesis and cancer cell proliferation, inducing apoptosis, and increasing immune response (Table 3). The enhancement of tumor response may contribute to the improvement of survival. Moreover, CMs may reduce the acute and subacute adverse reactions induced by TACE, thus improve the QoL. Further investigation on the therapeutic mechanism, pharmacokinetics, pharmacodynamics and their possible active components of the frequently used herbs could bring new insight into the treatment of HCC.

### 4.1. Limitations

Although extensive searches and strict methods were used to select studies and estimate the effects, there are several potential limitations. First, only studies published in English or Chinese were included, and studies published in other languages cannot be assessed. Second, as most studies were conducted among Chinese, the generalizability to other population needed to be further assessed. Third, clinical heterogeneity may be detected as CM preparations, dose, and treatment duration are varied across the included studies. Further studies are warranted to investigate the effects of different CM preparations in treating middle or late stage HCC. Fourth, sample size, selection criteria of subjects, and TACE drugs varied across the included studies, and the heterogeneity was not reflected in the data analyses. Fifth, most of the studies did not report the method of randomization, and all studies failed to report the method of allocation concealment and blinding (subjective outcomes), which might lead to the potential selection bias. Moreover, reasons for dropouts and withdrawals were mostly not described. Overall, these items were mostly at unclear or high risk of bias which could bias the findings of this review resulting in overestimation of the CMs beneficial effects.

### 4.2. Implications for Practice and Research

As most included studies have poor quality, future trials should be rigorously implemented using standard procedures following a standardized trial protocol (e.g., consolidated standards of reporting trials statement) [117, 118]. Another crucial issue is the quality control of CM preparations which consist of various CMs from different batches. As different properties of CMs might exist in different batches in the same CM formula, the quality control of further CM preparations should be established based on scientific practice including chemical and bioresponse fingerprint to ensure the quality and consistency of CM preparations and the validity of study results [119, 120]. In addition, CMs should be provided by a consistent and reliable supply to maintain the effective treatment effect of CM preparations. Given that oral administration and intravenous injection of CMs were used by all included studies, further reviews should compare the effects between these routes. Moreover, as no data on the possible interaction between TACE and CMs preparations was reported, the interaction should be assessed further. Only a small number of studies (33%) showed that the results of at least 12-month survival (the long-term effectiveness) of CMs treatment need to be determined in more RCTs with long-term follow-up. Acute and subacute CMs-related side effects in the studies were slight and alleviated spontaneously after symptomatic treatment. However, these were only reported in few studies (4%). Only short-term CMs-related side effects were reported. And all the side effects were not measured by standard criteria. Additional researches should evaluate both acute and chronic CMs-related side effects according to standard criteria to confirm the safety of CMs treatment in treating patients with HCC.

## 5. Conclusion

The positive results in this meta-analysis show that CMs treatment appears to increase the efficacy of TACE by prolonging survival, increasing tumor response, improving QoL, and reducing TACE toxicity for unresectable HCC. Although making a definitive recommendation is currently premature with low quality of the most studies, these findings suggest that CMs could be considered as an adjuvant therapy for unresectable HCC patients during TACE treatment. RCTs with rigorous methods, long-term follow-up, and standard reporting (consolidated standards of reporting trials statement) are recommended to further evaluate the clinical effects of combining CMs and TACE use for HCC patients [117, 118].

## Figures and Tables

**Table 1:** Characteristics of the included studies.

Study	Sample size	Design (sequence generation)	Baseline characteristics	Intervention		Duration	Outcome measures
	(T/C)			TACE	Experimental CMs		
Ayi and Liu 2011 [8]	108 (54/54)	Single centre, parallel group, unblinded RCT (unreported)	Mean age (range): 56 (28–77) Disease stage: NA Child-Pugh score: C KPS: >60	5-FU, HCPT, LP	Ai Di injection (60 L/d)	56 ds	(1) TR (short-term effectiveness) (2) Survival at 6/12/24 mons (3) KPS (QoL increase) (4) AE

Bao 2007 [9]	54 (28/26)	Single centre, parallel group, unblinded RCT (unreported)	Mean age (range): 51 (25–68) Disease stage: II, III Child-Pugh score: A, B KPS: NA	5-FU, DDP, MMC, HCPT, EPI, LP, GSP	Kang Ai injection (40–60 mL/d)	1 mon	(1) TR (short-term effectiveness) (2) AE

Cao et al. 2005 [10]	100 (50/50)	Single centre, parallel group, unblinded RCT (unreported)	Mean age (range): NA Disease stage: NA Child-Pugh score: A KPS: NA	5-FU, MMC, LP	Gan Fu Kang capsule (1 capsule, t.i.d.)	60–80 ds	(1) Survival at 6/12/24/36 mons

Chen and Ding 2007 [11]	60 (32/28)	Single centre, parallel group, unblinded RCT (random number table)	Age range: 36–70 Disease stage: NA Child-Pugh score: NA KPS: >60	5-FU, MMC, OX, LP, GSP	Ai Di injection (60 mL/d)	42 ds	(1) TR (short-term effectiveness) (2) KPS (QoL increase) (3) AE

Dan et al. 2007 [12]	70 (35/35)	Single centre, parallel group, unblinded RCT (sequence of admission)	Age range: 29–70 Disease stage: II, III, IV Child-Pugh score: NA KPS: NA	5-FU, DDP, THP, LP	Fu Zheng Ping Gan Xiao Liu Tang (1 dose/d)	1–6 mons	(1) TR (short-term effectiveness) (2) Survival at 6/12/18 mons (3) KPS (QoL increase)

Deng et al. 2009 [13]	40 (20/20)	Single centre, parallel group, unblinded RCT (unreported)	Mean age (range): 52 (26–66) Disease stage: III, IV Child-Pugh score: NA KPS: ≥70	THP, LP	Fu Fang Ku Shen injection (20 mL/d)	2 mons	(1) TR (short-term effectiveness) (2) KPS (QoL increase) (3) AE

Dong et al. 2007 [14]	65 (33/32)	Single centre, parallel group, unblinded RCT (unreported)	Mean age: 56.5 Disease stage: II, III, IV Child-Pugh score: NA KPS: ≥60	5-FU, DDP, THP, LP	Ai Di injection (80–100 mL/d)	56 ds	(1) TR (short-term effectiveness) (2) KPS (QoL increase) (3) AE

Dong et al. 2008 [15]	133 (67/66)	Single centre, parallel group, unblinded RCT (unreported)	Mean age (range): 56 (28–77) Disease stage: NA Child-Pugh score: NA KPS: >60	5-FU, THP, LP	Jing Long capsule (1 g, t.i.d.)	56 ds	(1) TR (short-term effectiveness) (2) Survival at 6/12/24 mons (3) KPS (QoL increase) (4) AE

Han 2009 [16]	48 (30/18)	Single centre, parallel group, unblinded RCT (unreported)	Mean age (range): 49.9 (32–70) Disease stage: II, III Child-Pugh score: NA KPS: NA	ADM, MMC, CBDCA, LP, GSP	Blood-activating and stasis-resolving herbs (NA)	NA	(1) TR (short-term effectiveness) (2) KPS (QoL increase)

Hou and Lu 2009 [17]	72 (36/36)	Single centre, parallel group, unblinded RCT (drawing of lots)	Age range: 34–72 Disease stage: NA Child-Pugh score: NA KPS: >70	DDP, BLM-A5, GC, LP, GSP	CMs given according to CM syndrome differentiation (1 dose/d)	4 wks	(1) TR (short-term effectiveness) (2) KPS (QoL increase) (3) AE

Huang et al. 2002 [18]	57 (30/27)	Multicentre, parallel group, unblinded RCT (unreported)	Mean age (range): 59.5 (35–70) Disease stage: II, III Child-Pugh score: NA KPS: NA	5-FU, MMC, HCPT, LP	Kang Lai Te injection (200 mL/d) plus Bai Hua She She Cao injection (30 mL/d)	2–4 mons	(1) TR (short-term effectiveness) (2) Survival at 12 mons (3) AE

Huang 2008 [19]	50 (30/20)	Single centre, parallel group, unblinded RCT (unreported)	Mean age (range): NA Disease stage: II, III Child-Pugh score: NA KPS: NA	5-FU, DDP, MMC, HCPT, LP	Ci Dan capsule (5 capsules, t.i.d.)	4 mons	(1) TR (short-term effectiveness) (2) Survival at 12 mons (3) AE

Jia et al. 2003 [20]	66 (34/32)	Single centre, parallel group, unblinded RCT (unreported)	Mean age (range): NA Disease stage: T3-4N0M0 Child-Pugh score: NA KPS: >60	5-FU, DDP, ADM, MMC, LP, GSP	*Brucea javanica* oil Injection (30 mL/d)	2–4 mons	(1) TR (short-term effectiveness) (2) AE

Li et al. 2009 [21]	64 (32/32)	Single centre, parallel group, unblinded RCT (unreported)	Mean age (range): NA Disease stage: II, III Child-Pugh score: NA KPS: ≥60	5-FU, DDP, ADM, LP	Kang Lai Te capsule (6 capsules, q.i.d.)	42–63 ds	(1) TR (short-term effectiveness) (2) AE

Li 2007 [22]	36 (20/16)	Single centre, parallel group, unblinded RCT (unreported)	Mean age (range): 50.9 (32–70) Disease stage: II, III Child-Pugh score: NA KPS: NA	MMC, THP, CBDCA, LP, GSP	CMs for fortifying the spleen and activating the blood (1 dose/d)	NA	(1) TR (short-term effectiveness) (2) KPS (QoL increase)

Li and Fan 2008 [23]	128 (64/64)	Single centre, parallel group, unblinded RCT (unreported)	Mean age (range): NA Disease stage: NA Child-Pugh score: NA KPS: >70	5-FU, EPI, MMC, LP	Fu Zheng Kang Ai Tang (1 dose/d)	3 mons	(1) TR (short-term effectiveness) (2) Survival at 6/12/24 mons (3) AE

Liang et al. 2005 [24]	68 (35/33)	Single centre, parallel group, unblinded RCT (unreported)	Age range: 29–70 Disease stage: II, III Child-Pugh score: NA KPS: NA	DDP, MMC, EPI, LP	Matrine injection (150 mL/d)	28 ds	(1) TR (short-term effectiveness) (2) AE

Liang et al. 2008 [25]	121 (64/57)	Single centre, parallel group, unblinded RCT (unreported)	Mean age (range): 44.8 (30–70) Disease stage: II, III Child-Pugh score: NA KPS: ≥60	5-FU, ADM, MMC, CBDCA, LP	Ci Dan capsule (5 capsules, q.i.d.)	2 mons	(1) TR (short-term effectiveness) (2) Survival at 6/12 mons (3) KPS (QoL increase)

Liang et al. 2005 [26]	146 (75/71)	Single centre, parallel group, unblinded RCT (random number table)	Mean age (range): 50.7 (20–74) Disease stage: III, IV Child-Pugh score: A, B, C KPS: NA	MMC, EPI, CBDCA, LP	Bu Zhong Yi Qi Tang (1st–3rd month: 1 dose/d; 4th–6th month: 2 doses/w)	6 mons	(1) TR (short-term effectiveness) (2) Survival at 6/12/24/36 mons

Ling 2010 [27]	128 (64/64)	Single centre, parallel group, unblinded RCT (unreported)	Age range: 39–62 Disease stage: II, III, IV Child-Pugh score: NA KPS: NA	5-FU, DDP, EPI, LP	Xiao Liu Tang (1 dose/d)	2-3 mons	(1) TR (short-term effectiveness) (2) KPS (QoL increase)

Liu et al. 2007 [28]	70 (34/36)	Single centre, parallel group, unblinded RCT (shuffling envelops)	Mean age (range): 50.7 (28–67) Disease stage: II, III Child-Pugh score: NA KPS: NA	5-FU, DDP, ADM, MMC, HCPT, LP, GSP	Kang Ai injection (40 mL/d)	20 ds	(1) TR (short-term effectiveness) (2) KPS (QoL increase) (3) AE

Lu and He 2009 [29]	48 (24/24)	Single centre, parallel group, unblinded RCT (unreported)	Age range: 28–68 Disease stage: II, III Child-Pugh score: NA KPS: NA	DDP, MMC, EPI, LP	Experience CMs formula (NA)	3–12 mons	(1) TR (short-term effectiveness) (2) KPS (QoL increase)

Lu et al. 2010 [30]	60 (30/30)	Single centre, parallel group, unblinded RCT (stratified randomization)	Mean age: 49.4 Disease stage: II, III, IV Child-Pugh score: NA KPS: NA	DDP, ADM, MMC, LP	Yang Gan Kang Ai Wan (9 g, t.i.d.)	135–270 ds	(1) Survival at 6/12/18 mons

Lu et al. 2007 [31]	63 (33/30)	Single centre, parallel group, unblinded RCT (unreported)	Age range: 18–71 Disease stage: II, III Child-Pugh score: A, B, C KPS: 50–90	DDP, GC, LP	Kang Ai injection (40 mL/d)	40 ds	(1) TR (short-term effectiveness) (2) KPS (QoL increase) (3) AE

Meng 2008 [32]	148 (75/73)	Single centre, parallel group, unblinded RCT (unreported)	Mean age: 56 Disease stage: II, III	5-FU, THP, LP	Ai Di injection (50 mL/d)	28 ds	(1) TR (short-term effectiveness) (2) Survival at 6/12/24 mons (3) KPS (QoL increase) (4) AE

Shi and Sun 2005 [33]	50 (30/20)	Single centre, parallel group, unblinded RCT (unreported)	Mean age (range): 52.3 (37–65) Disease stage: NA Child-Pugh score: NA KPS: NA	5-FU, MMC, LP, GSP	Tan Re Qing injection (40 mL/d)	≥14 ds	(1) KPS (QoL increase)

Qiao 2010 [34]	40 (20/20)	Single centre, parallel group, unblinded RCT (unreported)	Age range: 18–65 Disease stage: II, III Child-Pugh score: NA KPS: >50	5-FU, DDP, ADM, LP	Ai Tong Xiao granule (1 pack/d)	56 ds	(1) KPS (QoL increase) (2) AE

Sun et al. 2002 [35]	236 (118/118)	Single centre, parallel group, unblinded RCT (unreported)	Mean age (range): 51.4 (26–74) Disease stage: NA Child-Pugh score: NA KPS: NA	MMC, EPI, CBDCA, LP	Hua Chan Su injection (20 mL/d)	1–24 Ks	(1) Survival at 12/24/36 mons (2) AE

Tang et al. 2010 [36]	50 (30/20)	Multicentre, parallel group, unblinded RCT (unreported)	Mean age: 54.1 Disease stage: II, III Child-Pugh score: NA KPS: >60	ADM, MMC, LP, GSP	Fu Gan injection (20 mL/d)	2 mons	(1) TR (short-term effectiveness) (2) Survival at 6/12/24 mons (3) KPS (QoL increase)

Tian et al. 2001 [37]	43 (23/20)	Single centre, parallel group, unblinded RCT (unreported)	Mean age (range): 52.2 (23–73) Disease stage: II, III Child-Pugh score: NA KPS: 60–80	5-FU, DDP, ADM, LP	Fu Zheng Jie Du Tang (1 dose/d)	18–88 ds	(1) TR (short-term effectiveness) (2) KPS (QoL increase)

Tian 2006 [38]	72 (36/36)	Single centre, parallel group, unblinded RCT (unreported)	Mean age (range): 53 (33–75) Disease stage: NA Child-Pugh score: NA KPS: ≥70	5-FU, DDP, ADM, MMC, LP	Ai Yi Shu injection (0.5 mg/d)	NA	(1) TR (short-term effectiveness) (2) Survival at 6/12/18/24 mons (3) KPS (QoL increase) (4) AE

Wang et al. 2002 [39]	95 (47/48)	Multicentre, parallel group, unblinded RCT (unreported)	Mean age (range): 50.5 (28–73) Disease stage: NA Child-Pugh score: NA KPS: ≥60	5-FU, ADM, MMC, THP, HCPT, CBDCA, LP, GSP	960 mixture (NA)	42–210 ds	(1) TR (short-term effectiveness) (2) KPS (QoL increase) (3) AE

Wang and Cheng 2009 [40]	57 (27/30)	Single centre, parallel group, unblinded RCT (unreported)	Mean age (range): 48 (30–65) Disease stage: II, III Child-Pugh score: NA KPS: ≥70	5-FU, DDP, ADM, LP, GSP	Fu Fang Ku Shen injection (20 mL/d)	≥20 ds	(1) TR (short-term effectiveness) (2) Survival at 12/24/36 mons (3) KPS (QoL increase)

Wang and Yang 2002 [41]	60 (30/30)	Single centre, parallel group, unblinded RCT (unreported)	Mean age: 55.7 Disease stage: II, III Child-Pugh score: NA KPS: >60	ADM, DDP, MMC, LP	Gan Ji granule (1 pack, b.i.d.)	3-4 mons	(1) TR (short-term effectiveness) (2) Survival at 6/12 mons

Wang et al. 2008 [42]	86 (43/43)	Single centre, parallel group, unblinded RCT (unreported)	Mean age: 55.56 Disease stage: II, III Child-Pugh score: NA KPS: >60	DDP, ADM, MMC, LP	Jian Pi Qing Gan He Ji (200 mL, b.i.d.)	2 mons	(1) TR (short-term effectiveness)

Wang et al. 2007 [43]	43 (22/21)	Single centre, parallel group, unblinded RCT (random number table)	Mean age (range): 64 (35–78) Disease stage: II, III Child-Pugh score: NA KPS: NA	ADM, MMC, CBDCA	Qi Shu Fang (1 dose/d)	56 ds	(1) TR (short-term effectiveness) (2) KPS (QoL increase) (3) AE

Wang 2008 [44]	59 (30/29)	Single centre, parallel group, unblinded RCT (unreported)	Mean age (range): 61 (38–78) Disease stage: NA Child-Pugh score: NA KPS: NA	5-FU, ADM/EPI, MMC, LP, GSP	Experience CMs formula (1 dose/d)	>2 mons	(1) TR (short-term effectiveness) (2) KPS (QoL increase)

Weng et al. 2008 [45]	96 (55/41)	Single centre, parallel group, unblinded RCT (unreported)	Age range: 28–76 Disease stage: NA Child-Pugh score: A, B KPS: NA	5-FU, DDP, ADM, MMC, LP, GSP	Experience CMs formula plus CM patch (NA)	NA	(1) TR (short-term effectiveness) (2) Survival at 6/12/24/36/48/ 60 mons (3) KPS (QoL increase) (4) AE

Wu et al. 2000 [46]	80 (36/44)	Single centre, parallel group, unblinded RCT (unreported)	Mean age: 51.4 Disease stage: II, III Child-Pugh score: NA KPS: NA	5-FU, DDP, MMC, LP, GSP	Hua Chan Su injection (20 mL/d)	20 ds	(1) TR (short-term effectiveness) (2) Survival at 6/12 mons (3) AE

Wu 1999 [47]	25 (13/12)	Single centre, parallel group, unblinded RCT (unreported)	Mean age (range): 55.4 (38–69) Disease stage: II Child-Pugh score: NA KPS: NA	5-FU, DDP, MMC, LP	Yi Guan Jian Jia Wei (NA)	24 wks	(1) Survival at 36 mons

Wu et al. 2003 [48]	60 (30/30)	Single centre, parallel group, unblinded RCT (unreported)	Mean age (range): 51.9 (28–72) Disease stage: II, III	5-FU, ADM (or DDP), MMC, LP, GSP	Hu Gan Ruan Jian Fang (NA)	NA	(1) TR (short-term effectiveness) (2) Survival at 6/12 mons

Xu et al. 2006 [49]	57 (30/27)	Single centre, parallel group, unblinded RCT (sequence of admission)	Mean age (range): 52.3 (39–72) Disease stage: NA Child-Pugh score: NA KPS: NA	5-FU, MMC, HCPT, LP	Fu Zheng Jie Du Tang (1 dose/d)	2–4 mons	(1) TR (short-term effectiveness) (2) Survival at 12 mons

Xu et al. 2007 [50]	52 (32/20)	Single centre, parallel group, unblinded RCT (unreported)	Age range: 38–75 Disease stage: II, III Child-Pugh score: NA KPS: NA	5-FU, DDP, THP, LP	Ai Di Injection (50 mL/d)	4 wks	(1) TR (short-term effectiveness) (2) KPS (QoL increase) (3) AE

Xu et al. 2007 [51]	60 (40/20)	Single centre, parallel group, unblinded RCT (unreported)	Age range: 35–72 Disease stage: II, III Child-Pugh score: NA KPS: NA	5-FU, DDP/OX, HCPT, LP	CMs for fortifying the spleen and resolving dampness and activating the blood and detoxifying (1 dose/d)	2 mons	(1) TR (short-term effectiveness) (2) AE

Xue et al. 2002 [52]	70 (34/36)	Single centre, parallel group, unblinded RCT (unreported)	Mean age (range): 51.3 (26–72) Disease stage: III, IV Child-Pugh score: NA KPS: NA	5-FU, DDP, ADM, MMC, LP	Si Jun Zi Tang (1 dose/d)	NA	(1) TR (short-term effectiveness) (2) Survival at 12/24/36 mons (3) AE

Yang 2010 [53]	50 (25/25)	Single centre, parallel group, unblinded RCT (unreported)	Mean age (range): 54 (31–74) Disease stage: NA Child-Pugh score: A, B KPS: NA	5-FU, DDP, ADM, EPI, LP	Lian Hua Qing Gan Yin (1 dose/d)	NA	(1) Survival at 12/24 mons

Yang 2006 [54]	62 (31/31)	Single centre, parallel group, unblinded RCT (unreported)	Age range: 27–68 Disease stage: NA Child-Pugh score: NA KPS: NA	5-FU, DDP, EPI, LP, GSP	Ai Di injection (50 mL/d)	>1 mon	(1) TR (short-term effectiveness) (2) KPS (QoL increase) (3) AE

Yang 2006 [55]	50 (28/22)	Single centre, parallel group, unblinded RCT (unreported)	Mean age (range): 50.6 (26–75) Disease stage: II, III, IV Child-Pugh score: NA KPS: >60	5-FU, DDP, MMC, THP, LP	Ai Di injection (50 mL/d)	32 ds	(1) TR (short-term effectiveness) (2) AE

Yi et al. 2008 [56]	67 (36/31)	Single centre, parallel group, unblinded RCT (shuffling envelops)	Mean age (range): 53.9 (25–69) Disease stage: II, III Child-Pugh score: NA KPS: >60	5-FU, ADM, HCPT, LP, GSP	Kang Ai injection (40 mL/d)	12 wks	(1) TR (short-term effectiveness) (2) KPS (QoL increase) (3) AE

Yu and Kang 2010 [57]	96 (48/48)	Multicentre, parallel group, unblinded RCT (unreported)	Mean age (range): 53.1 (30–69) Disease stage: II, III Child-Pugh score: NA KPS: NA	5-FU, ADM, HCPT, LP, GSP	Fu Fang Ku Shen injection (20 mL/d)	45 ds	(1) TR (short-term effectiveness) (2) KPS (QoL increase) (3) AE

Yuan et al. 2010 [58]	62 (31/31)	Multicentre, parallel group, unblinded RCT (unreported)	Mean age (range): NA Disease stage: NA Child-Pugh score: NA KPS: NA	5-FU, DDP, MMC, THP, LP, GSP	CMs for soothing the liver, fortifying the spleen, and tonifying the kidney (NA)	3 mons	(1) TR (short-term effectiveness) (2) KPS (QoL increase)

Yuan and Yu 2005 [59]	73 (35/38)	Single centre, parallel group, unblinded RCT (unreported)	Age range: 34–69 Disease stage: NA Child-Pugh score: NA KPS: >50	5-FU, DDP, MMC, HCPT	Ai Di injection (50 L/d)	>20 ds	(1) TR (short-term effectiveness)

R. Q. Zhai and H. Y. Zhai 2010 [60]	62 (32/30)	Single centre, parallel group, unblinded RCT (random number table)	Mean age (range): 55.3 (28–72) Disease stage: III, IV Child-Pugh score: NA KPS: ≥50	MMC, EPI, CBDCA, LP	Hu Gan Xiao Zheng Tang (1st–3rd month: 1 dose/d; 4th month: 1 dose/2 d) plus San Jie Xiao Tong Gao (plaster therapy; 1 dose/2 ds)	4 mons	(1) TR (short-term effectiveness) (2) Survival at 6/12/24/36 mons (3) KPS (QoL increase) (4) AE

Zhang et al. 2005 [61]	224 (116/108)	Single centre, parallel group, unblinded RCT (unreported)	Mean age (range): 51.3 (29–76) Disease stage: NA Child-Pugh score: NA KPS: NA	MMC, EPI, CBDCA, LP	Jing Long capsule (4 capsules, t.i.d.)	≥3 yrs	(1) TR (short-term effectiveness) (2) Survival at 6/12/24/36 mons

Zhang et al. 2007 [62]	60 (30/30)	Single centre, parallel group, unblinded RCT (unreported)	Mean age (range): 50 (24–75) Disease stage: II, III Child-Pugh score: NA KPS: ≥60	5-FU, DDP, ADM, MMC, LP, GSP	Chai Shao Liu Jun Zi Tang (1 dose/d)	>1 mon	(1) TR (short-term effectiveness) (2) KPS (QoL increase) (3) AE

Zhang et al. 2008 [63]	61 (31/30)	Single centre, parallel group, unblinded RCT (stratified randomization)	Mean age (range): 50.2 (24–67) Disease stage: II, III Child-Pugh score: A, B, C KPS: NA	5-FU, MMC, THP, LP	CMs given according to CM syndrome differentiation (1 dose/d)	≥2 mons	(1) TR (short-term effectiveness) (2) Survival at 6/12/18/24 mons (3) KPS (QoL increase)

Zhang et al. 2008 [64]	64 (31/33)	Single centre, parallel group, unblinded RCT (sequence of admission)	Age range: 39–73 Disease stage: II, III, IV Child-Pugh score: NA KPS: >60	5-FU, DDP, HCPT, OX, LP	Jian Pi Fu Gan Tang (1 dose/d)	>2 mons	(1) TR (short-term effectiveness) (2) Survival at 6/12/18 mons (3) KPS (QoL increase)

Zhang et al. 2007 [65]	112 (58/54)	Single centre, parallel group, unblinded RCT (random number table)	Mean age (range): 57.2 (18–70) Disease stage: II Child-Pugh score: NA KPS: ≥50	5-FU, DDP, EPI, VDS	Gu Ben Yi Liu II (NA)	≥2 mons	(1) TR (short-term effectiveness) (2) Survival at 6/12/24 mons (3) KPS (QoL increase) (4) AE

Zhang 2011 [66]	49 (25/24)	Single centre, parallel group, unblinded RCT (unreported)	Mean age (range): 49.9 (33–71) Disease stage: II, III Child-Pugh score: NA KPS: >60	NA	Blood-activating and stasis-resolving herbs (NA)	2-3 mons	(1) TR (short-term effectiveness) (2) KPS (QoL increase)

Zhang et al. 2000 [67]	95 (50/45)	Multicentre, parallel group, unblinded RCT (unreported)	Age range: 29–60 Disease stage: II, III Child-Pugh score: NA KPS: NA	5-FU, DDP/ADM, MMC, LP	CMs for soothing the liver and regulating Qi, fortifying the spleen and harmonizing the stomach, tonifying the liver and kidney, and softening hardness and dissipating binds (1 dose/d)	≥2-3 mons	(1) TR (short-term effectiveness) (2) Survival at 6/12/24 mons

Zhao and Huang 2005 [68]	60 (30/30)	Single centre, parallel group, unblinded RCT (unreported)	Age range: 38–72 Disease stage: II Child-Pugh score: NA KPS: NA	5-FU, EPI, HCPT, LP	Can Qi capsule (NA)	NA	(1) TR (short-term effectiveness) (2) AE

Zhao et al. 2006 [69]	94 (48/46)	Single centre, parallel group, unblinded RCT (sequence of admission)	Mean age (range): 52.8 (40–64) Disease stage: II, III Child-Pugh score: NA KPS: >70	5-FU, DDP, ADM, HCPT, LP, GSP	Liu Jun Zi Tang (1 dose/d)	126–168 ds	(1) TR (short-term effectiveness) (2) Survival at 12/24/36 mons

Zhou et al. 2002 [70]	228 (118/110)	Single centre, parallel group, unblinded RCT (sequence of admission)	Age range: 28–72 Disease stage: II, III Child-Pugh score: NA KPS: NA	5-FU, DDP, ADM, MMC, LP, GSP	Liu Jun Zi Tang (1 dose/d)	NA	(1) Survival at 6/12/24/36 mons

Zhou et al. 2010 [71]	64 (32/32)	Single centre, parallel group, unblinded RCT (unreported)	Mean age (range): 49.7 (22–70) Disease stage: NA Child-Pugh score: A, B KPS: ≥60	MMC, BLM-A5, LP, GSP	Kang Ai Fang (1 dose/d)	3 mons	(1) TR (short-term effectiveness) (2) Survival at 12 mons (3) KPS (QoL increase)

Zou 2004 [72]	50 (25/25)	Single centre, parallel group, unblinded RCT (unreported)	Mean age: 41 Disease stage: II, III Child-Pugh score: NA KPS: NA	5-FU, MMC, HCPT, LP	Fu Fang Ku Shen injection (16 mL/d)	40 ds	(1) TR (short-term effectiveness) (2) AE

5-Fu: fluorouracil; AE: TACE toxicity; b.i.d.: two times a day; BLM-A5: bleomycin A5; C: control group; CBDCA: carboplatin; CM: Chinese medicine; d: day; DDP: cisplatin; EPI: epirubicin; GC: gemcitabine; HCPT: hydroxycamptothecin; KPS: Karnofsky performance scale; MMC: mitomycin; mon: month; NA: not available; OX: oxaliplatin; q.i.d.: four times a day; QoL: quality of life; T: treatment group; TACE: transarterial chemoembolization; THP: pirarubicin; t.i.d.: three times a day; TR: tumor response; VDS: vindesine; wk: week; yr: year.

**Table 2:** Risk of bias assessment.

Study	Sequence generation	Allocation concealment	Blinding: outcomes				Blinding	Incomplete outcome data	Selective outcome data	Other bias	Risk of bias score
			Survival	Tumor response	KPS	AE					
Ayi and Liu 2011 [8]	U	U	Y	Y	/	Y	Y	Y	U	Y	3
Bao 2007 [9]	U	U	/	Y	/	N	N	U	U	Y	1
Cao et al. 2005 [10]	U	U	Y	/	/	/	Y	Y	Y	Y	4
Chen and Ding 2007 [11]	Y	U	/	Y	N	N	N	Y	U	Y	3
Dan et al. 2007 [12]	N	U	Y	Y	N	/	N	Y	Y	U	2
Deng et al. 2009 [13]	U	U	/	Y	N	/	N	Y	Y	U	2
Dong et al. 2007 [14]	U	U	/	Y	N	/	N	Y	U	U	1
Dong et al. 2008 [15]	U	U	Y	Y	N	Y	N	N	U	Y	1
Feng 2002 [73]	U	U	/	Y	/	/	Y	Y	U	Y	3
Guo et al. 2005 [74]	U	U	/	Y	/	N	N	U	Y	U	1
Han 2009 [16]	U	U	/	/	N	/	N	N	Y	Y	2
Hou and Lu 2009 [17]	Y	U	/	Y	N	N	N	U	Y	Y	3
Huang et al. 2002 [18]	U	U	Y	Y	/	N	N	Y	U	U	1
Huang 2008 [19]	U	U	Y	Y	/	N	N	Y	U	U	1
Jia et al. 2003 [20]	U	U	/	Y	/	N	N	Y	Y	Y	3
Li et al. 2009 [21]	U	U	/	Y	/	N	N	N	Y	Y	2
Li 2007 [22]	U	U	/	Y	N	/	N	U	Y	U	1
Li and Fan 2008 [23]	U	U	Y	Y	/	N	N	Y	U	Y	2
Liang et al. 2005 [24]	U	U	/	Y	/	Y	Y	U	Y	Y	3
Liang et al. 2008 [25]	U	U	Y	Y	N	/	N	U	Y	Y	2
Liang et al. 2005 [26]	Y	U	Y	Y	/	/	Y	U	Y	Y	4
Ling 2010 [27]	U	U	/	Y	N	/	N	Y	Y	U	2
Liu et al. 2007 [28]	Y	U	/	Y	N	N	N	Y	Y	Y	4
Lu and He 2009 [29]	U	U	/	Y	N	/	N	Y	Y	Y	3
Lu et al. 2010 [30]	Y	U	Y	/	/	/	Y	Y	Y	Y	5
Lu et al. 2007 [31]	U	U	/	Y	N	N	N	Y	Y	Y	3
Meng 2008 [32]	U	U	Y	Y	N	Y	N	Y	U	Y	2
Qiao 2010 [34]	U	U	/	/	N	N	N	U	Y	Y	2
Shi and Sun 2005 [33]	U	U	/	/	N	/	N	Y	U	Y	2
Sun et al. 2002 [35]	U	U	Y	/	/	N	N	U	U	Y	1
Tang et al. 2010 [36]	U	U	Y	Y	N	/	N	U	Y	Y	2
Tian et al. 2001 [37]	U	U	/	Y	N	/	N	U	Y	Y	2
Tian 2006 [38]	U	U	Y	Y	N	U	N	Y	Y	Y	3
Wang et al. 2002 [39]	U	U	/	Y	N	N	N	U	Y	Y	2
Wang and Cheng 2009 [40]	U	U	Y	Y	N	/	N	Y	Y	Y	3
Wang and Yang 2002 [41]	U	U	Y	Y	/	/	Y	Y	U	Y	3
Wang et al. 2008 [42]	U	U	/	Y	/	/	Y	Y	U	Y	3
Wang et al. 2007 [43]	Y	U	/	Y	N	Y	N	Y	Y	Y	4
Wang 2008 [44]	U	U	/	Y	N	/	N	Y	Y	U	2
Weng et al. 2008 [45]	U	U	Y	Y	N	U	N	Y	Y	Y	3
Wu et al. 2000 [46]	U	U	Y	Y	/	N	N	Y	Y	Y	3
Wu 1999 [47]	U	U	Y	/	/	/	Y	U	U	U	1
Wu et al. 2003 [48]	U	U	Y	Y	/	/	Y	U	U	Y	2
Xu et al. 2006 [49]	N	U	Y	Y	/	/	Y	U	U	U	1
Xu et al. 2007 [50]	U	U	/	Y	N	N	N	Y	Y	Y	3
Xu et al. 2007 [51]	U	U	/	Y	/	N	N	U	U	Y	1
Xue et al. 2002 [52]	U	U	Y	Y	/	Y	Y	Y	Y	Y	4
Yang 2010 [53]	U	U	Y	/	/	/	Y	U	N	Y	2
Yang 2006 [54]	U	U	/	Y	N	N	N	Y	Y	U	2
Yang 2006 [55]	U	U	/	Y	/	Y	Y	U	U	U	1
Yi et al. 2008 [56]	Y	U	/	Y	N	N	N	U	N	Y	2
Yu and Kang 2010 [57]	U	U	/	Y	N	Y	N	U	N	Y	1
Yuan et al. 2010 [58]	U	U	/	Y	N	/	N	Y	Y	Y	3
Yuan and Yu 2005 [59]	U	U	/	Y	/	/	Y	Y	U	U	2
R. Q. Zhai and H. Y. Zhai 2010 [60]	Y	U	Y	Y	N	N	N	Y	Y	Y	4
Zhang et al. 2005 [61]	U	U	Y	Y	/	/	Y	N	U	Y	2
Zhang et al. 2007 [62]	U	U	/	Y	N	N	N	Y	Y	Y	3
Zhang et al. 2008 [63]	Y	U	Y	Y	N	/	N	Y	Y	Y	4
Zhang et al. 2008 [64]	N	U	Y	Y	N	/	N	Y	U	Y	2
Zhang et al. 2007 [65]	Y	U	Y	Y	N	Y	N	Y	Y	Y	4
Zhang 2011 [66]	U	U	/	Y	N	/	N	U	Y	Y	2
Zhang et al. 2000 [67]	U	U	Y	Y	/	/	Y	Y	U	Y	3
Zhao and Huang 2005 [68]	U	U	/	Y	/	/	Y	Y	Y	Y	4
Zhao et al. 2006 [69]	N	U	Y	Y	/	/	Y	U	U	U	1
Zhou et al. 2002 [70]	N	U	Y	/	/	/	Y	Y	Y	U	3
Zhou et al. 2010 [71]	U	U	Y	Y	N	/	N	N	U	Y	1
Zou 2004 [72]	U	U	/	Y	/	N	N	Y	Y	U	2

AE: transarterial chemoembolization toxicity; KPS: Karnofsky performance scale. Y: yes; N: no; U: unclear.

**Table 3:** The top 10 most frequently used CMs of the included studies.

CM herb Latin name (Chinese Pinyin)	No.	TCM diagnosis	Pharmacological properties
Radix Astragali (Huang Qi)	35	Qi deficiency	(1) Suppresses the oncogenic transformation of cancer cells [86] (2) Induces apoptosis [87] (3) Induces macrophage, LAK and NK cell activity [88, 89] (4) Inhibits T-helper cell type 2 cytokines [89]

Poria Cocos (Fu Ling)	25	Dampness accumulation	(1) Induces apoptosis [90, 91] (2) Cytotoxicity against cancer cell lines [90] (3) Inhibits tumor angiogenesis [92]

Rhizoma Atractylodis Macrocephalae (Bai Zhu)	23	Qi deficiency	(1) Induces apoptosis [93, 94]

Radix Ginseng (Ren Shen)	19	Qi deficiency	(1) Induces apoptosis [95, 96] (2) Inhibits tumor cell proliferation [96] (3) Cytotoxicity against cancer cell lines [97, 98] (4) Inhibits tumor angiogenesis [99]

Radix Bupleuri (Chai Hu)	19	Qi stagnation	(1) Induces apoptosis [100] (2) Activates macrophages, NK and LAK cells [101] (3) Downregulates TNF-*α*, IL-6, and NF-*κ*B p65 expression [102]

Radix Codonopsis (Dang Shen)	18	Qi deficiency	(1) Inhibits cancer cells invasion and migration [103] (2) Enhances T cell, B cell, and macrophage production, and activates macrophages [104]

Semen Coicis (Yi Yi Ren)	15	Dampness accumulation	(1) Induces apoptosis [105] (2) Inhibits NF-*κ*B signaling and protein kinase C activity [106] (3) Stimulates T cell proliferation [107]

Herba Oldenlandia Diffusa (Bai Hua She She Cao)	14	Fire toxin	(1) Inhibits cancer cell proliferation and induces apoptosis [108, 109]

Radix Paeoniae Alba (Bai Shao)	13	Blood deficiency	(1) Inhibits angiogenesis and induces apoptosis [110, 111]

Rhizoma Curcumae (E Zhu)	12	Blood stagnation	(1) Inhibits cancer cell proliferation and angiogenesis, induces cell cycle arrest and apoptosis [112] (2) Inhibits platelet aggregation [113]

CM: Chinese medicine; LAK: lymphokine activated killer; NF-*κ*B: nuclear factor kappa-light-chain enhancer of activated B cells; NK: natural killer; No.: number of studies; TCM: traditional Chinese medicine; TNF-*α*: tumor necrosis factor-alpha; IL: interleukin.

## Abbreviations

ALT: Alanine transaminase
CBM: Chinese biomedical CD database
CIs: Confidence intervals
CMs: Chinese medicines
CMCC: Chinese medical current contents
CNKI: China network knowledge infrastructure
CR: Complete response
HCC: Hepatocellular carcinoma
KPS: Karnofsky performance scale
MD: Mean difference
PR: Partial response
PRISMA: Preferred reporting items for systematic reviews and meta-analyses
QoL: Quality of life
RCT: Randomized controlled trial
RRs: Risk ratios
TACE: Transarterial chemoembolization
CM: Chinese medicine
CMs: Chinese medicines
WHO: World health organization.
